# Identification of BcARR Genes and CTK Effects on Stalk Development of Flowering Chinese Cabbage

**DOI:** 10.3390/ijms23137412

**Published:** 2022-07-03

**Authors:** Xi Ou, Yudan Wang, Jiewen Zhang, Zhenbin Xie, Bing He, Zhehao Jiang, Yuting Wang, Wei Su, Shiwei Song, Yanwei Hao, Riyuan Chen

**Affiliations:** College of Horticulture, South China Agricultural University, Guangzhou 510642, China; ouxi13272462926@163.com (X.O.); ydwang@stu.scau.edu.cn (Y.W.); hnzyzij@stu.scau.edu.cn (J.Z.); xj-99gzxwz@stu.scau.edu.cn (Z.X.); binhe@stu.scau.edu.cn (B.H.); zhjiang@stu.scau.edu.cn (Z.J.); wangyt@163.com (Y.W.); susan_l@scau.edu.cn (W.S.); swsong@scau.edu.cn (S.S.)

**Keywords:** flowering Chinese cabbage, type-B ARR, cytokinin signaling, GA signaling, bolting

## Abstract

Flowering Chinese cabbage (*Brassica campestris* L. ssp. *Chinensis* var. utilis Tsen et Lee) is an important and extensively cultivated vegetable in south China, whose major food product is the stalk. In the process of stalk formation, its initiation and development are regulated by a series of hormonal signals, such as cytokinin and gibberellin. In this study, we analyzed the effects of zeatin (ZT) and gibberellin A3 (GA3), and their interaction, on the bolting of flowering Chinese cabbage. The results indicated that the three-true-leaf spraying of ZT and GA synthesis inhibitor (PAC) inhibited plant height but increased stem diameter. Cytokinin (CTK) synthesis inhibitor (YZJ) and GA3 treatment increased plant height and decreased stem diameter. In addition, ZT and GA3 co-treated plants displayed antagonistic effect. Further, 19 type-B authentic response regulators (ARR-Bs), the positive regulators of cytokinin signal transduction were identified from flowering Chinese cabbage. Comprehensive analysis of phylogeny showed BcARR-Bs clustered into three subfamilies with 10 conserved motifs. Analysis of their expression patterns in different tissues and at various growth stage, and their response to hormone treatment suggest that ARR1-b localized in the nucleus displayed unique highest expression patterns in stem tips, are responsive both to ZT and GA, suggesting a significant role in mediating the crosstalk of ZT and GA in the bolting of flowering Chinese cabbage.

## 1. Introduction

Flowering Chinese cabbage (*Brassica campestris* L. ssp. *Chinensis* (L.) Makino var. *utilis* Tsen et Lee) is planted throughout the worldwide, as a subspecies of Chinese Cabbage, which originated in south China. The edible part of flowering Chinese Cabbage is the stalk, the development of which consist of bolting (stem elongation and thickening) and flowering. The product quality and yield depend on both of them. Factors that affect the timing of the bolting of flowering Chinese Cabbage include temperature and plant hormones [1,2,3]. Exogenous gibberellin A3 (GA3) treatment advances bolting time, stem elongation, and flowering time. In addition, cytokinin is important phytohormone that plays vital roles throughout plant growth and development [4,5,6,7,8]. GA and cytokinin exert antagonistic effects on numerous developmental processes, including shoot and root elongation, cell differentiation and meristem activity [9,10]. Moreover, several recent studies have shown reciprocal interactions between the two hormones where cytokinin inhibits the production of GA and promotes its deactivation and GA inhibits cytokinin responses [10,11,12,13]. In Medicago truncatula (Fabaceae), GA signaling decreases the amount of bioactive cytokinin in roots and negatively impacts the cytokinin-dependent symbiotic nodulation [14].

Cytokinin signal transduction is a multistep phosphorelay, similar to bacterial two-component response systems [15]. Using this system, plants respond to various environmental changes, including temperature, light period, and nutrient supply to regulate a variety plant process [7,16,17,18]. The two-component system incorporates histidine (His) protein Kinases, His-containing phosphotransferases (Hpts), and responses regulators (RRs) [19]. On the basis of the protein structure and conserved amino acid domains, response regulators (RRs) are classified into two distinct types, type-A and type-B. Type-B ARRs contain a conserved receiver domain in their N-terminal region. The C-terminal extensions are of variable length and possess a DNA binding Myb-like domain, which is responsible for both nuclear localization and activation [20]. Owing to truncated C-terminal domains constitutively activate cytokinin responses, the receiver domain is thought to inhibit transcriptional activity of the type-B ARR [21,22].

The type-B ARR family members are transcription factors that function as positive regulators of cytokinin signal transduction, resulting in rapid induction of cytokinin-associated target genes, including the type-A ARR genes, which are negative regulators of cytokinin [15]. In addition, a previous study had shown type-B ARR would recruit DELLA proteins that are negative regulators of GA signaling and co-regulate the downstream target genes during root meristem growth and photomorphogenesis [23], indicating its potential role in mediating the crosstalk of cytokinin and gibberellin in the process of the bolting and flowering of flowering Chinese cabbage.

In this study, we investigated the effects of ZT, CTK synthesis inhibitor (YZJ), GA3 and GA synthesis inhibitor (PAC) on plant height, stem diameter, hormone content and cell morphology in flowering Chinese Cabbage. In addition, we analyzed the gene structures, chromosome distribution, phylogenetic relationships, conserved motifs and cis-regulatory element of all putative BcARR-B genes. In order to further understand the role of BcARR-B in these processes, we also conducted the analysis of the expression profiles of ARR-B genes in different tissues and under different hormones treatments. The comprehensive analysis of BcARR-B will provide substantial useful information for studying these BcARR-B transcription factors in the bolting of flowering Chinese cabbage.

## 2. Results

### 2.1. Effect of Exogenous Hormones Treatment on the Bolting of Flowering Chinese Cabbage ‘Youlv501’

**Figure 1:**
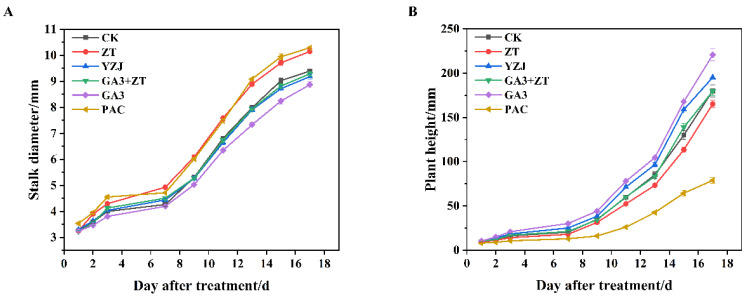
Effect of plant hormones spraying on the growth of flowering Chinese cabbage. (**A**) Stalk diameter. (**B**) Plant height. We measured the stalk thickness and plant height at 1 day after treatment. The units on the lower axis are the number of days after treatment in (**A**,**B**). The data represent an average of three replicates ± standard error.

To analyze the effect of stalk development in flowering Chinese cabbage in response to exogenous hormones treatments, we treated the plants at three-true-leaf stages with ZT, YZJ, GA3, and PAC. The stem diameter and plant height were calculated. As shown in Figure 1A,B, the effect of PAC and ZT treatment on stem diameter and plant height is antagonistic to that of the GA3 and YZJ treatment. PAC and ZT-treated plants appeared to be thicker and shorter than that of the control. The plant height of YZJ-treated plants was slightly shorter than that of GA3 treated plants. In addition, the GA3 + ZT treatment had no effect on the stem diameter and plant height showing no significant difference compared with control, indicating an antagonistic effect on the stem development between GA3 and ZT.

### 2.2. Effect of Exogenous Hormones Spraying on the Cell Size in Shoot Apices

**Figure 2:**
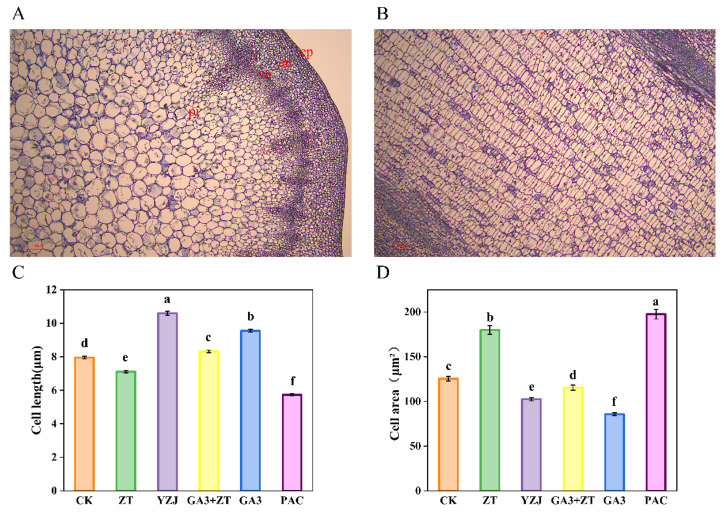
Histological analysis of shoot apices in hormones-treated flowering Chinese cabbage at bud emergence stages. (**A**,**B**) Microscopic images of transverse and longitudinal sections of shoot apices: ep, epidermis; en, endodermis; va, vascular bundle; pi, pith cells. (**C**) Length of pith cells in longitudinal sections of shoot apices. (**D**) Cell area of pith cell in transverse sections of shoot apices. Statistically significant differences in mean values at different sampling points are indicated by different letters (Duncan’s test, *p* < 0.05). Bars are SE (*n* = 4).

As CTK is reported to regulate cell division and cell differentiation during plant development, the increased diameter may result from the increased cell growth and cell expansion. To examine this possibility, we conducted histological analyses of the shoot apices of flowering Chinese cabbage under different hormones at bud emergence as shown in Figure 2. Compared with control, ZT and PAC treatment decreased the cell length of pith cells in longitudinal section of shoot apices, whereas the cell area of pith cells in transverse section of shoot apices of the ZT and PAC treated-plants were bigger at bud emergence stage. However, GA3 and YZJ treatment decreased cell area of pith cells in transverse section and promoted the elongation growth of pith cells in longitudinal section of shoot apices at bud emergence stage. In addition, at bud emergence stage, GA3 and ZT exert antagonistic effects on pith cell size.

### 2.3. Effect of Exogenous Plant Hormones Spraying on the Hormone Content in Shoot Apices

**Figure 3:**
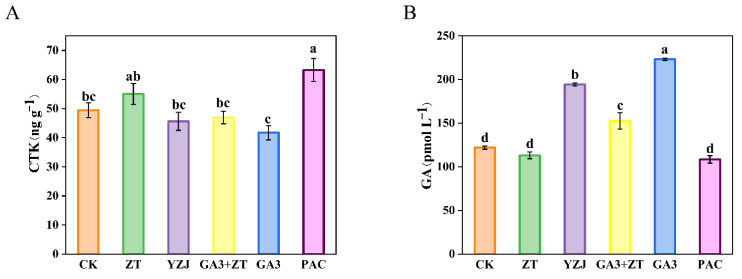
Hormone contents in shoot apices of flowering Chinese cabbage under control and different hormone treatments. (**A**) cytokinin (CTK). (**B**) gibberellin (GA). Statistically significant differences in mean values at different sampling points are indicated by different letters (Duncan’s test, *p* < 0.05). Bars are SE (*n* = 4).

As the bolting was regulated by plant hormones, we measured the content of CTK and GA in the shoot apices of flowering Chinese cabbage under different exogenous hormones treatment at bud emergence stage. As shown in Figure 3, the content of CTK was enhanced by ZT and PAC treatment at bud emergence stage, whereas GA3 treatment reduced the content of CTK at the same stages, which can be rescued by spraying of exogenous ZT. For GA content, we observed different results: YZJ and GA3 treatment enhanced the GA content at bud emergence stage. In addition, ZT and PAC treatment reduced the GA content but without the significant difference. When GA3 and ZT synchronously treated the flowering Chinese cabbage, ZT would counteract the GA3-induced promotion of GA content. 

### 2.4. qRT-PCR Analysis of Bolting-Related Genes Responded to ZT in Flowering Chinese Cabbage

**Figure 4:**
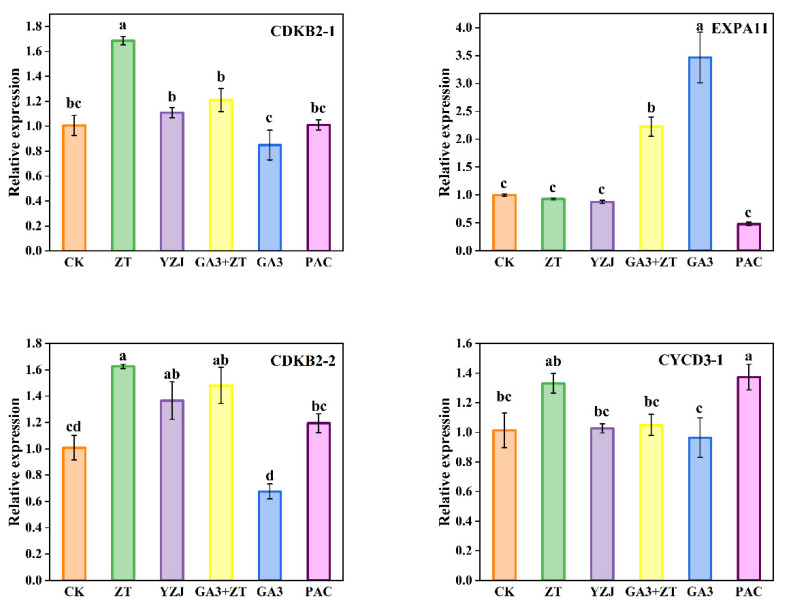
Expression profiles of flowering elongation and cyclin genes in flowering Chinese cabbage. Data represent the mean ± standard error for three biological experiments, with standard errors shown as bar charts above the columns, while lowercase letters indicate significance at *p* < 0.05.

Three cyclin-related genes—CDKB2-1, CDKB2-2 and CYCD3-1—and one cell expansion gene—EXPA11—were selected to analyze the expression characteristics of these genes at bud emergence stage after different hormones. EXPA11 functions as a key factor responsible for cell expansion by promoting the sliding of polymers such as cell wall cellulose and hemicellulose [24,25]. In addition, cell cycle regulation affects stem elongation and thickening in the shoot meristem [26]. CDK and CYCD play important role in G1 to S progression [27]. CYCD3-1 is a D-type plant cyclin gene through which cytokinin activates cell division [28]. As shown in Figure 4, at bud emergence stage, the ZT treatment increased the expression level of CDKB2-1, CDKB2-2 and CYCD3-1, whereas GA3 treatment showed opposite effect. Moreover, GA3-treated and GA3 + ZT-cotreated plants show higher expression level of EXPA11, which was similar to findings in a previous study [29]. In addition, PAC treatment also increased the expression level of CYCD3-1. 

### 2.5. Identification, and Analysis of the BcARR Genes

**Figure 5:**
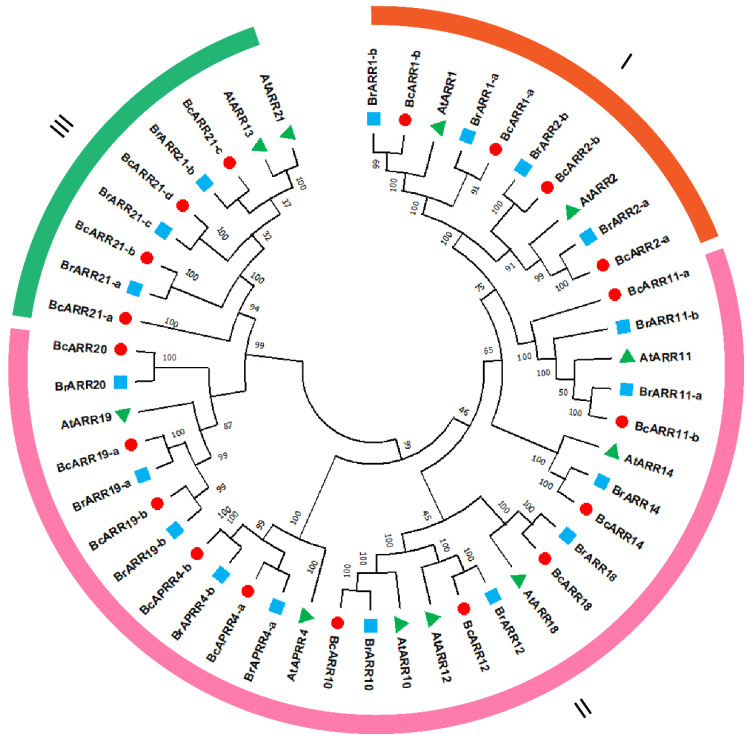
Phylogenetic analysis of the type-B ARR families in *Arabidopsis thaliana*, *Brassica rapa*, and *Brassica campestris*. A neighbor-joining phylogenetic tree was constructed from 48 protein sequences from *Arabidopsis thaliana* (11), *Brassica rapa* (18) and *Brassica campestris* (19). The tree was divided into three subfamilies (subfamilies I, II, and III).

**Figure 6:**
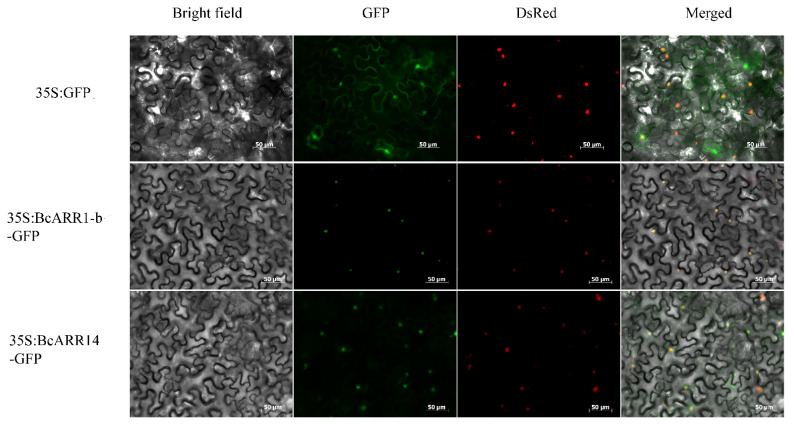
Subcellular localization of BcARR1-b and BcARR14 in *Nicotiana benthamiana*. BcARR1-b and BcARR14 were fused to N-terminus of the hGFP. BcARR1-b/BcARR14-GFP fusion proteins were transiently expressed in leaves of *Nicotiana Benthamiana* and subcellular localization was analyzed by microscopy. The merged pictures of green fluorescence channel (middle panels), the red fluorescence channel (middle panels) and the corresponding bright field (left panel) are shown (right panels). The scale bar indicates 50 μm. The nuclei were stained with DsRed.

As the type-B authentic response regulators (ARR-Bs) function as positive regulators of cytokinin signal transduction and may play a role in mediating the crosstalk of cytokinin and gibberellin, we identified the ARR-Bs genes in flowering Chinese cabbage. In the present study, 19 candidate B-ARR genes were obtain for *B. campestris*. The results of sequence analysis exhibited that the length of BcARR amino acid residue ranged from 287 aa (Bra-cxA07g010490.1) to 895 aa (Bra-cxA02g 003110.1), isoelectric point (pI) values ranged from 5.13 (Bra-cxA 09g01397.1) to 9.5 (Bra-cxA03g064180.1) and molecular weights ranged from 32.79 kDa (Bra-cxA07g010490.1) to 98.01kDa (Bra-cxA02g003110.1) (Supplementary Table S5). To further investigate the evolutionary relationship of B-ARR proteins in *A. thaliana*, *B. rapa*, and *B. campestris*, a rootless neighbor-joining phylogenetic tree was constructed with 1000 bootstrap replicates using the full-length sequences of the proteins. The proteins were named according to the phylogenetic and their orthology with the reported isoform in *Arabidopsis*. The family was divided into three subfamilies. ARR1-a, ARR1-b, ARR2-a, and ARR2-b were clustered into subfamily I; ARR21-a, ARR21-b, ARR21-c and ARR21-d belong to subfamily II, and the others were ascribed to subfamily III (Figure 5). The subcellular location was analyzed with WoLF PSORT, which showed that the majority of BcARR-Bs are nuclear proteins, except for BcARR1-a (chloroplast), and BcARR11-a (cytosol) (Supplementary Table S5). BcARR1-b and BcARR14 were chosen to confirm the subcellar localization, the CDS of them lacking the stop codon was fused to the N-terminus of the hGFP reporter gene and ligated into an expression vector under a confocal microscope. The BcARR1-b and BcARR14-GFP fusion protein localized to nucleus, which indicated that BcARR1-b and BcARR14-GFP is functional protein in nucleus (Figure 6). 

### 2.6. Gene Structure and Protein Motifs of BcARR Genes

**Figure 7:**
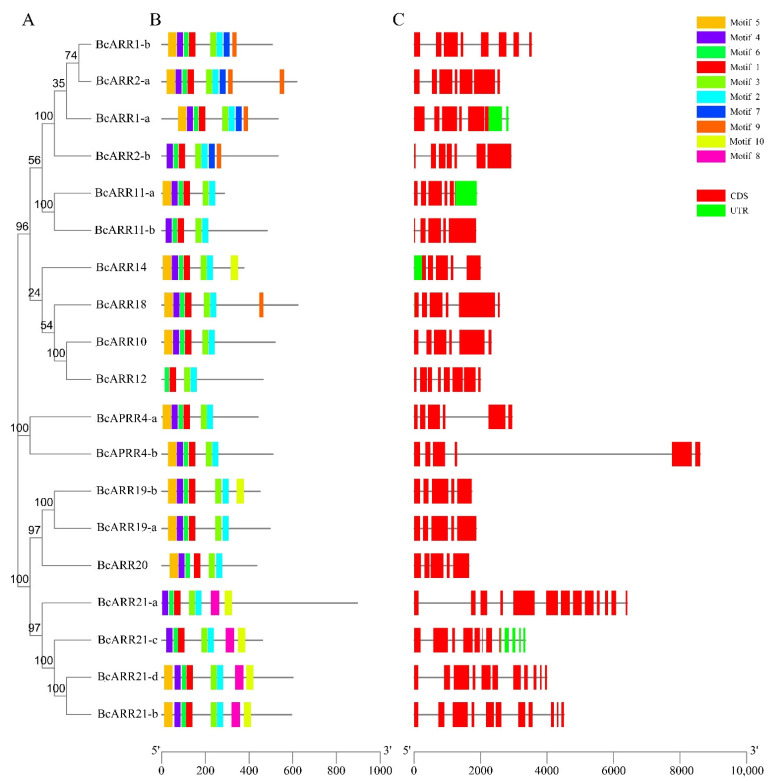
Phylogenetic relationships, gene structure and motif analysis of BcARR-B genes, (**A**) Phylogenetic tree of BcARR-B genes. (**B**) Distribution of conserved motifs in BcARR-B genes; different colors represent the 10 conserved domain identified. (**C**) Gene structure of BcARR-B genes, where red boxes represent CDS and green boxes represent UTR. The lengths of the boxes and lines are scaled according to the length of the genes.

The B-ARR gene family was found to be diverse in the number of introns and exons with the number of exons varying from 4 to 13, and the number of introns ranging from 4 to 12. BcAPRR4-b had extremely long intron (Figure 7). Using the NCBI-CDD database and MEME program, the conserved domains and motifs were further analyzed, respectively. We found B-ARR proteins were highly conserved on the N-terminal region, which was consistent with a previous report [30]. The type-B ARRs contain a phosphorylatable REC domain and a large C-terminal extension with a Myb-like DNA binding domain referred to as the GARP domain [20,30]. A total of 10 widely-distributed conserved motifs were identified (Figure 4). All ARR-B genes were clustered into three subfamilies (I–III) based on the compositions of conserved motif by introducing Brassica rapa and Arabidopsis thaliana ARR-B genes. Motif 1, Motif 2, Motif 3, and Motif 6 were present in all BcARR proteins, with Motif 1 containing 30 amino acids, Motif 2 containing 29 amino acids, Motif 3 containing 28 amino acids, and Motif 6 containing 21 amino acids. In addition, Motif 4 and Motif 5 were also present in majority of the ARR proteins, containing 29 and 40 amino acids, respectively. Meanwhile, Motif 8 only exists in the subfamily III, both Motif 7 and Motif 9 exist in subfamily I. These motifs represent conserved motifs and functional domains.

### 2.7. Chromosomal Distribution and Collinearity Analysis of BcARR Genes

**Figure 8:**
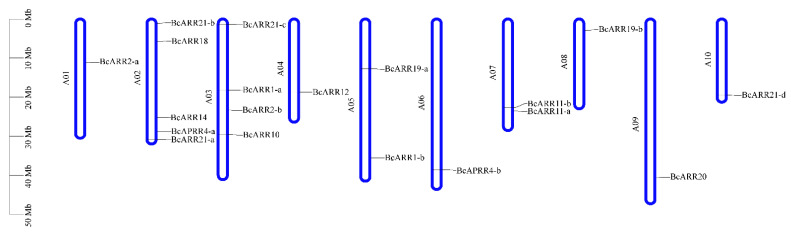
Chromosomal distribution of 19 BcARR-B genes in flowering Chinese cabbage.

**Figure 9:**
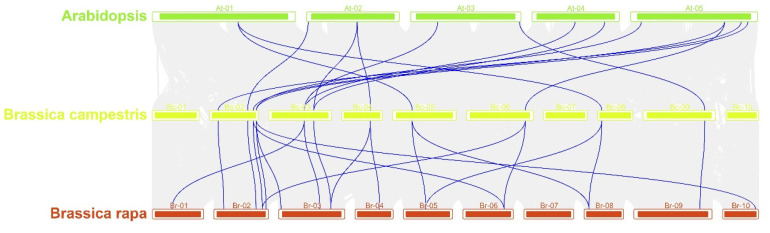
Collinearity of type-B ARR among *Brassica campestris*, *Brassica rapa*, and *Arabidopsis thaliana*. Blue lines indicate the syntenic gene pairs.

We mapped the chromosomal locations of the B-ARR genes in *B. campestris*, and the distribution of the genes on each chromosome was uneven, with most genes located on A02 (5 genes) and A03 (4 genes), followed by chromosome A05 and A07, which contained 2 genes, respectively. Only one gene was located on A01, A04, A08, A09, and A10 (Figure 8). The collinear relationships of the B-ARR gene pairs among *A. thaliana*, *B. rapa*, and *B. campestris* are shown in Figure 9; the B-ARRs in *B. campestris* and *B. rapa* possessed a closer collinear relationship (Figure 9).

### 2.8. Analysis of Cis-Acting Elements in the Promoter Regions of B-ARR Genes

**Figure 10:**
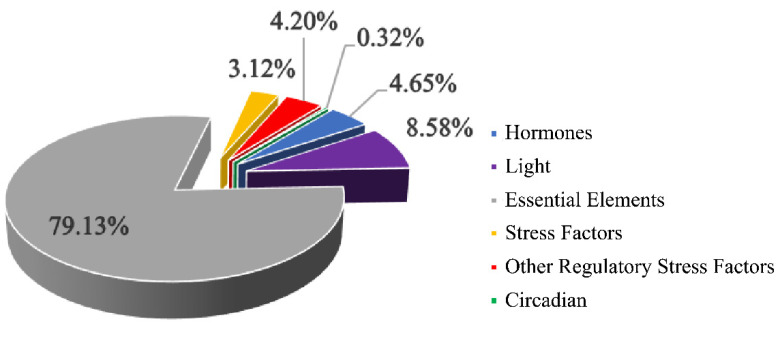
The ratios of different cis-elements.

The cis-elements in promoter regions are closely associated with gene transcription. Therefore, 2.0 Kb upstream sequence were obtained from *B. campestris* genome, and analyzed using the Plant CARE database [31]. We identified a total of six common cis-regulatory elements (Supplementary Table S4). As shown in Figure 10, these genes contained the largest number of essential elements, including CAAT box and TATA box, which are the core elements of the promoter in eukaryotes [32,33]. In addition, few of the cis-regulatory elements were actively receptive against hormone, such as gibberellin, auxin, salicylic acid, abscisic acid, and MeJA. Among them, gibberellin-responsive elements including GARE motif, P-box and TATC-box were located in the promoter regions of ARR1-b, APRR4-a, ARR11-a, ARR11-b, ARR12, ARR18, ARR19-a and ARR21-a, indicating that the expression of these BcARR-B genes were responsive to gibberellin.

### 2.9. Expression Patterns of the BcARR Genes in Flowering Chinese Cabbage

**Figure 11:**
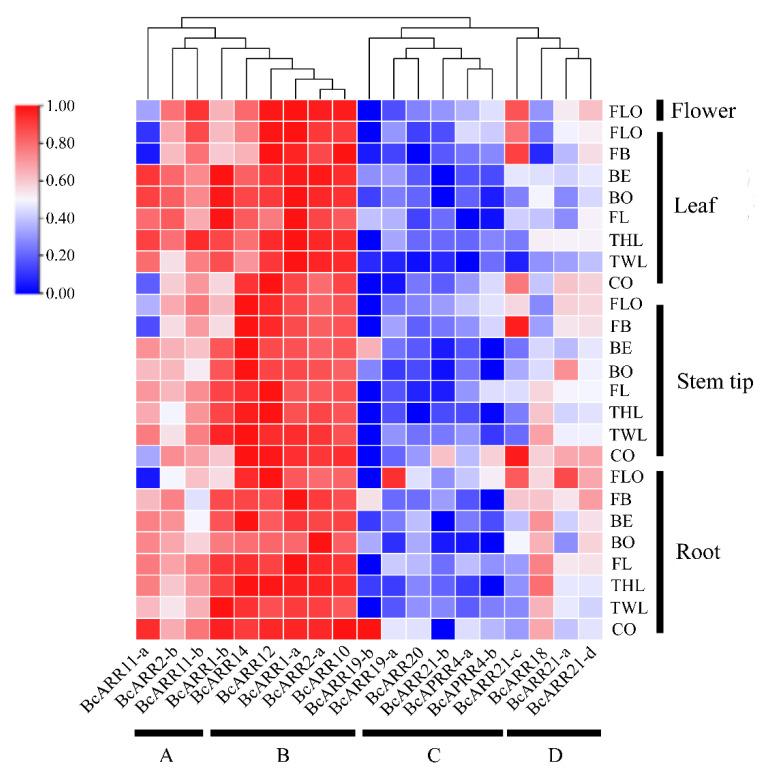
Heatmap of BcARR-B genes transcribed in the tissues of flowering Chinese cabbage at different development stages. CO, cotyledon stage; TWL, two-leaf stage; THL, three-leaf stage; FL, four-leaf stage; BO, bolting stage; BE, bud emergence stage; FB, fast bolting stage; FLO, flowering stage.

To preliminarily investigate the functional roles of ARR-B in flowering Chinese cabbage developmental process, by using RT-PCR, the expression patterns of flowering Chinese cabbage ARR-B were analyzed in different tissue at seven stages including cotyledon, two-leaf, three-leaf, four-leaf, bolting, bud emergence, fast bolting and flowering stage (Figure 11). Based on the expression level, ARR-Bs genes were clustered into four groups. Group A including ARR11-a, ARR2-b, ARR11-b showed modulate expression level in the root, stem tip, leaf and flower tissues all through the seven stages. Group B contains ARR1-a, ARR1-b, ARR2-a, ARR10, ARR12 and ARR14 showing the highest expression level in these four tissues tested during the whole development stages, indicating that these ARR-B genes have great influence on various processes of flowering Chinese cabbage growth and development. While Group C and Group D showed weak expression level in all these tissues.

### 2.10. Effect of Exogenous Hormones Spraying on Expression of BcARR Genes

**Figure 12:**
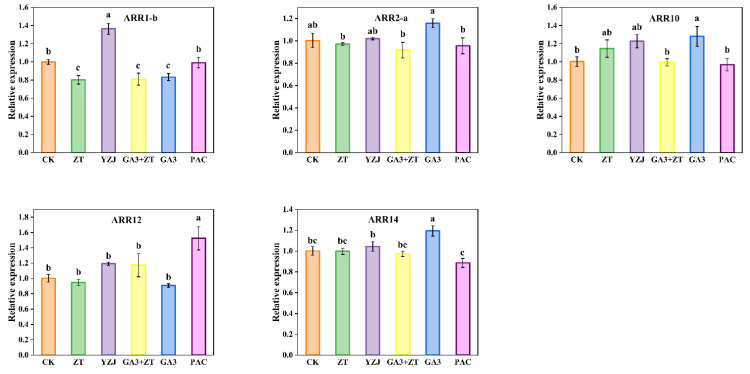
Five BcARR-B genes were analyzed by qRT-PCR expression in stem tip of flowering Chinese cabbage at bud emergence stage under different hormone treatments. Data represent the mean ± standard error for three biological experiments, with standard errors shown as bar charts above the columns, while lowercase letters indicate significance at *p* < 0.05.

To further understand the function of Group B ARR-B genes with highest expression except for ARR1-a with chloroplast-localized, we conducted RT-PCR using the materials mentioned above. BcARR-B genes showed diverse responses to the different hormones. As shown in Figure 12, at bud emergence stage, ZT treatment inhibited the expression level of ARR1-b, and YZJ treatment showed an opposite effect on the ARR1-b. ZT treatment had no significant influence on the other ARR-B genes. Moreover, PAC treatment significantly increased the expression level of ARR12 and slightly inhibited ARR14 expression level. In addition, GA3 treatment increase the majority of these ARR-B genes except for ARR1-b and ARR12 at bud emergence stage. Overall, only ARR1-b could both respond to CTK and GA, suggesting its important role in mediating the crosstalk of these two hormones.

## 3. Discussion

Cytokinin and GA are two classes of plant hormones that regulate many developmental and physiological process in plants. Cytokinin treatment results in a reduction in hypocotyl elongation of wild-type seeding [34]. Furthermore, cytokinin deficiency reduced the activity of the shoot meristems and caused the formation of an enhanced root system [8]. The mutation and overexpression of multiple cytokinin metabolism IPT and CKX genes in *Arabidopsis* and poplar resulted in changes in diameter of the root and stem [8,35]. In addition, cytokinin increased vegetative growth period by suppressing florigen expression in rice and maize [36]. GA and cytokinin exert antagonistic effects on numerous developmental processes, including shoot and root elongation, cell differentiation and meristem activity [9,10]. Moreover, several recent studies have shown reciprocal interactions between the two hormones where cytokinin inhibits the production GA and promotes its deactivation and GA inhibits cytokinin responses [11]. In flowering Chinese cabbage, endogenous plant hormones activities are closely relayed to bolting [3,24]. Three-true-leaf spraying of cytokinin promoted the thickening growth of diameter and inhibited stem elongation. However, GA3 treatment had opposite effect on plant height and stem diameter which was similar to a previous study [3]. ZT and GA3 cotreated-plants displayed antagonistic effect on stem diameter and plant height. Meanwhile, the CTK and GA content in the shoot apices showed that ZT and PAC treatment reduced GA content while GA and YZJ treatment decreased CTK content, indicating ZT and GA3 regulates stalk development interdependently. A regulator mediating these two hormones during the stalk development needs to be clarified.

In plants, the cytokinin signal is perceived by membrane-located sensor histidine kinases [37,38]. Additionally, a multistep phosphorelay system transmits the signal to type-B response regulator proteins via phosphotransmitter proteins. It has been reported that ARR-Bs function as positive regulators in cytokinin signal transduction, which involved in plant growth and environmental stress [39,40]. The ARR1, ARR2, ARR10 triple mutant exhibited increasement in hypocotyl elongation, reduction in length and wide of leaves and reduced width of inflorescence stems [34,41]. Furthermore, mutations in type-B ARRs (ARR1, ARR2, ARR10, ARR12) result in defects in shoot regeneration and axillary meristem development [42]. ARR2 has also been proposed to play a significant role in mediating ethylene response [43]. In rice and maize, cytokinin affect flowering time by suppressing florigen expression by inhibiting Ehd1, a type-B response regulator [36]. Moreover, it is reported that DELLA proteins would be recruited to type-B ARRs, which enhance their transactivation ability and co-regulate the downstream target genes including the CKX3 in the CTK metabolite [23].

Considering the potential role of ARR-B in mediating the crosstalk of these two hormones, we analyzed the type-B ARR gene family in *Brassica campestris*, which included 19 numbers. By analyzing the intron-exon structure of BcARR-B genes, we found that they have different number of introns. According to the compositions of conserved motifs, all these ARR-B genes were classified into three distinct subfamilies by introducing *Brassica rapa* and *Arabidopsis thaliana* ARR-B genes. All of these genes contained ARR-B phosphorylatable REC domain and Myb-like DNA binding domain, consistent with results in other plants such as Arabidopsis, tobacco and tomato [44,45,46]. The conserved domains are implicated in an early step in the cytokinin signal transduction and activation of downstream target genes [15]. The number of species harboring type-B ARR family gene in brassica campestris was higher than the number in Arabidopsis. This may be the result of gene duplication. In plant, the evolution and amplification of gene family were attributed to gene duplication, which is believed as a fundamental driving force in the evolution of genomes [46,47,48,49]. The expression data showed that all of the duplicated type-B gene pairs (ARR2-a and ARR2-b) exhibited diverse expression patterns, suggesting that functional diversification of the duplicated genes is a major feature of evolution [50].

The GARP domain of ARR10 contains a helix–loop–helix structure, suggesting that ARR-Bs are possibly nuclear-localized transcription factors [41]. Based on the expression profiles, we obtained six predominantly expressed genes in distinct tissue at diverse stages including ARR1-a, ARR1-b, ARR2-a, ARR10, ARR12 and ARR14, whereas subcellular location analysis showed that ARR1-a protein might be located in chloroplast. Therefore, the other five BcARR-B genes were selected for further analysis. The elements analysis showed that gibberellin-responsive elements were found located in the promoter of ARR1-b and ARR12. Meanwhile, at bud emergence stage, ZT treatment caused the down-regulation of the expression of ARR1-b gene, increased the expression of CDKB2-1, CDKB2-2 and CYCD3-1 which play an important role in G1 to S progression. In a previous study, RNA-seq of flowering Chinese cabbage showed that the expression level of ARR1-b was upregulated by cold treatment at the bolting stage [51]. In addition, cold treatment alters the contents of CTK [3]. Moreover, ZT treatment induced the expression level of RGA1 and RGL1 which played key role in the stem growth and early bud differentiation, respectively [52] (Figure S1). Besides showing positive effect on the expression of ARR1-b, GA3 treatment up-regulated the expression of ARR10 and ARR14 and caused the down-regulation of CDKB2-1, CDKB2-2, CYCD3-1, RGA1 and RGL1. GA3 treatment also significantly upregulated the transcript level of EXPA11 similar to previous studies [29]. Only ARR1-b both response to ZT and GA, indicating its role in the crosstalk of CTK and GA. The edible parts of flowering Chinese cabbage are stalk, whose size is influenced by two determinants, cell number and size. Therefore, we hypothesized that ZT and GA spraying caused the transcript change in type-B ARR and altered cell division and cell lateral expansion and ultimately modified the stem thickening and plant height.

## 4. Materials and Methods

### 4.1. Identification of Type-B ARR Genes in Flowering Chinese Cabbage

To identify type-B ARR genes in Brassica campestris, Arabidopsis type-B ARR protein sequences (Supplementary Table S1) were used as queries to carry out HMM search against the Brassica campestris whole genome by using BLASTP program. The NCBI-CDD (https://www.ncbi.nlm.nih.gov/Structure/cdd/wrpsb.cgi, accessed on 18 December 2022) database [53] was used to eliminate genes that did not contain the known conserveed domains and motifs. Finally, the final sequence file was manually selected for follow-up experiment. The ExPASy online tool (https://web.expasy.org/compute_pi/, accessed on 18 December 2022) was used to calculate the basic physic -chemical properties including molecular weight (MW) and isoelectric point (pI) [54]. WoLF PSORT (https://www.genscript.com/wolf-psort.html?src=leftbar, accessed on 18 December 2022) was used to predict the subcellular location of BcARR proteins [55]. 

### 4.2. Chromosomal Location, Multiple-Sequence Alignment, and Phylogenetic Analysis

Chromosomal position mapping of BcARR genes was performed using TBtools software (TBtools_JRE1.6, Guangzhou, China) [56]. All the BcARR sequences were aligned using ClustalW, a neighbor-joining phylogenetic tree was constructed, with Poisson correction, pairwise deletion and bootstrap (1000 replicates; random seeds), as parameters, utilizing MEGA11 [57]. For the phylogenetic tree of type-B ARR proteins from *Arabidopsis thaliana*, *Brassica rapa*, and *Brassica campestris*, the complete ARR protein sequences were used with methods previously described. The type-B ARR protein sequences of *A. thaliana* and *B. rapa* were obtained from the Arabidopsis Information Resource Archive database and the Brassica database (BRAD2) separately [58].

### 4.3. Synteny Analysis

Toolkits of TBtools software were used to carry out synteny analysis [56]. Syntenic blocks and distinct duplication events were identified by One Step MCScanX, and the synteny relationships of the orthologous ARR genes between the Brassica campestris and the other selected two representative plant species were displayed by Dual Synteny Plot.

### 4.4. Analysis of Gene Structure and the ARR Motif

The MEME tools (version 5.4.1) was used to identified the conserved motifs in the BcARR sequence with the following parameters: site distribution, zero-or-one-site-per-sequence (ZOOPS) model; the maximum number of motifs was set to 10, and the optimal motif width was 6 to 50 amino acid residues [59]. All obtained motifs and exon-intron structural information were visualized using the Gene Structure View (Advanced) of the TBtools software [56]. 

### 4.5. Promoter Sequence Analysis

In this stage, 2000 bp upstream sequences of the transcriptional start site of each BcARRs were chosen to identify the cis-elements in the putative promoter regions of the BcARRs. The PlantCARE website (http://bioinformatics.psb.ugent.be/webtools/plantcare/html/, accessed on 18 December 2022) was used to identify the putative cis-regulatory elements along the promoter sequences [31].

### 4.6. Plant Growth, Sample Preparation and Treatments

Flowering Chinese cabbage cultivar ‘*Youlv501*’ was obtained from Guangzhou Academy of Agricultural Science and planted in pots with perlite and cultivated in the greenhouse of college horticulture at the South China Agriculture University.

The ‘Youlv501’ flowering Chinese cabbage was used as the experimental material. The seeds were surface-sterilized in 7.5% NaClO for 10 min, soaked in distilled water for 2 min, and washed three times to remove NaClO. Seeds were germinated on five layers of wet filter paper in a glass Petri dish and placed in a plant incubator (22/18 °C with 16 h light/8 h dark cycle, light intensity 300 mol m^−2^·s^−1^) for one day. In the exogenous-spraying treatment, when the young seedlings were at the three-true-leaf stage, plants were transplanted into 15 cm diameter seedling pots filled with matrix pearlite and coconut (volume: volume: volume, 3:1:1), and sprayed with ZT (40 mg/L), YZJ (40 mg/L), GA3 (200 mg/L) and uniconazole (10 mg/L). Shoots (including the stem tip, root and leaf tissue) were collected from both the treated and control plants at, the bolting stage, the bud stage, rapid bolting stage and the flowering stage. Three biological replicates were used for each condition, and twenty seedlings were used for each biological replicate. All samples were immediately frozen in liquid nitrogen and stored at −80°.

### 4.7. Determination of Characteristic Related to Stem Development

The plant height and stem diameter of 4th internode of the flowering Chinese cabbage were measured by ruler and vernier caliper. The microscopic characteristic related stem development such as cell lengths in longitudinal sections, cell area in transverse sections and the number of cells were measured by the Image J software (Image-Pro Plus 6.0).

### 4.8. Histological Analysis

The stem tips (5 mm) were collected and fixed in formaldehyde alcohol acetic acid solution (70% alcohol:acetic acid:formaldehyde = 90:5:5), placed under a vacuum for 20 min, and incubated for 48 h at 4 °C. The samples were then dehydrated in a gradient of ethanol and embedded in paraffin [60]. Subsequently, 8 mm-thick sections were stained with reddish-green stain and observed under a microscope. Approximately 80 to 100 cells were randomly selected to calculate the cell size using Image-Proplus 6.0. Each treatment was conducted in three replicates.

### 4.9. RNA Extraction, Gene Cloning and Expression Analysis through qRT-PCR

Total RNA was isolated using the HiPure RNA Mini Kit (Magen, Guangzhou, China) following the manufacturer’s instructions and removing genomic DNA. Total RNA was quantified spectrophotometrically by measuring the A260/A280 and A260/A230 ratios, respectively, using a Nanodrop (Thermo Fisher Scientific, Waltham, MA, USA) and 2% *w*/*v* agarose gel. First-strand cDNA was synthesized using the Hiscript QRT SuperMix (with gDNA Wiper; Vazyme, Nanjing, China) in a reaction volume of 20 µL according to the instruction. Coding sequences (CDS) lacking the stop codon of BcARR1-b and BcARR14 isolated from the shoot apex were cloned using gene-specific primers (Supplementary Table S2). The qRT-PCR was performed on a Bio-Rad CFX384 TouchTM system using the SYBR^®^ Green Premix Pro Taq HS qRT-PCR Kit (Accurate Biotechnology, AG11718, Hunan, China) with primers provided in Supplementary Table S3. The qRT-PCR reaction condition was as follows: 95 °C for 5 min; then 39 cycles at 95 °C for 10 s and 60 °C for 30 s, followed by 65–95 °C melting curve detection. The primers for qRT-PCR were designed using the Primer 3.0 with melting temperatures of 58–62 °C, GC% of 40.0–60.0, and amplification product size of 80–200 bp, and synthesized by Guangzhou Tianyi Biotech Company (Guangzhou, China). The qRT-PCR efficiency of the genes was obtained by analyzing the standard curve of cDNA gradient dilution, and the housekeeping gene Actin2/GADPH was used as the internal control to normalize the mount of template cDNA. Relative expression values for each gene were computed using the comparative 2^−ΔΔCT^ method with normalization to the internal control gene [61]. The TBtools software was used to visualize Gene expression profiles as a heatmap [56].

### 4.10. Quantification of Hormone Content

Approximately 50 mg of shoot apex tissue was harvested from each plant. To the dissected tissue, 450 µL of 1× phosphate-buffered saline was added. The tissue was ground with a mortar and pestle, and then homogenized by sonication. The homogenate was centrifuged to pellet cellular debris and the supernatant was transferred to a clean tube for ELISA. The CTK content of the tissue samples was analyzed using a plant Cytokinin (CTK) ELISA Kit (Shanghai Enzyme-Linked Biotechnology Co., Ltd, Guangzhou, China) following the manufacturer’s protocol. The O.D. of the samples is measured spectrophotometrically at a wavelength of 450 nm. The concentration of CTK is then determined by comparing the O.D. of the samples to the standard curve. The same protocol was used for GA measurements.

### 4.11. Subcellular Localization Analysis

The full-length cDNA sequence of BcARR1 was fused to the N-terminus of the hGFP gene with expression driven by the CaMV 35S promoter. The plasmid and localization signal (NLS-DsRed) was transformed into *Agrobacterium tumefaciens* strain GV3101 and then introduced into *Nicotiana benthamiana* leaves with an injection method [62]. GFP expression in different subcellular compartments was detected at 448 nm and 550 nm, respectively, by laser scanning confocal microscopy (Axioimager. D2) after 24 h at 22 °C in darkness, as described elsewhere [29]. Three biological replicates were performed in this experiment.

## 5. Conclusions

Taken together, the results of present study suggest CTK may affect bolting by decreasing plant height and increasing stem diameter through affecting pith cell size and cell cycle gene expression level. During this process, GA functions antagonistically with CTK. We identified 19 BcARR-B genes from *Brassica campestris* and positioned them unevenly on 10 chromosomes. Based on the structure of motif, these BcARR-B proteins were classified into three subfamilies. Four conserved motifs are widely present in the proteins. Five key genes (ARR1-b, ARR2-a, ARR10, ARR12, and ARR14) showed high expression levels on different tissue at eight stages. Under CTK and GA treatment, only ARR1-b responds to both of the two hormones, indicating an important role in mediating the crosstalk of these hormones in the bolting of flowering Chinese cabbage.

## Data Availability

All important data is included in the article and Supplementary Material.

## Supplementary Materials

The following are available online at https://www.mdpi.com/article/10.3390/ijms23137412/s1.
