# Supplementary material for: Identification of BcARR Genes and CTK Effects on Stalk Development of Flowering Chinese Cabbage
Source: Int J Mol Sci. 2022 Jul 3;23(13):7412. doi: 10.3390/ijms23137412 (PMC9266762; doi:10.3390/ijms23137412)
Supplement: Supplementary file 1 [file ijms-23-07412-s001.zip › supplementary figures.pdf]

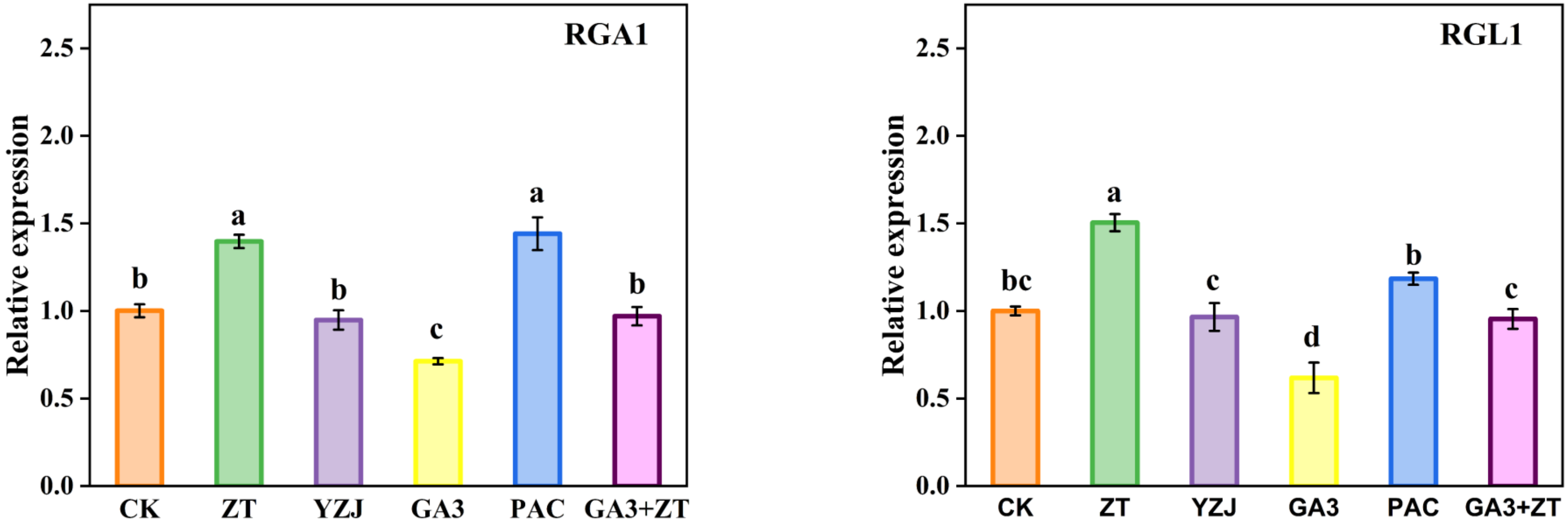

Figure S1. Expression profiles of flowering elongation and cyclin genes in flowering Chinese cabbage. Data represent the mean ± standard error for three biological experiments, with standard errors shown as bar charts above the columns, while lowercase letters indicate significance at $p < 0.05$.
