# Supplementary material for: Identification of BcARR Genes and CTK Effects on Stalk Development of Flowering Chinese Cabbage
Source: Int J Mol Sci. 2022 Jul 3;23(13):7412. doi: 10.3390/ijms23137412 (PMC9266762; doi:10.3390/ijms23137412)
Supplement: Supplementary file 1 [file ijms-23-07412-s001.zip › Table S5.pdf]

Table S5. Physicochemical properties of *type B ARR* genes in flowering Chinese cabbage.

Gene	CDS （bp）	AA	pI	MW （KD）	Subcellular localization
BcARR19-b	1350	450	5.27	51.2	nucl: 12, chlo: 1, vacu: 1
BcARR19-a	1488	496	6.07	56.0	nucl: 9, cyto: 2, pero: 2, chlo: 1
BcARR20	1308	436	5.13	50.0	nucl: 10.5, cyto_nucl: 6.5, cyto: 1.5, pero: 1, cysk: 1
BcARR1-b	1518	506	5.85	54.9	nucl: 9, chlo: 2, plas: 2, cysk: 1
BcARR14	1128	376	9.21	41.5	nucl: 7, cyto: 4, mito: 1, plas: 1, cysk: 1
BcARR1-a	1596	532	9.04	59.0	chlo: 8, nucl: 4, plas: 1, E.R.: 1
BcARR2-a	1854	618	6.07	67.5	nucl: 14
BcARR11-a	861	287	8.74	32.8	cyto: 6, chlo: 2, nucl: 2, mito: 2, plas: 1, extr: 1
BcARR10	1557	519	5.92	58.0	nucl: 11, cyto: 3
BcARR2-b	1599	533	8.64	59.5	nucl: 6, mito: 6, chlo: 2
BcARR18	1872	624	5.33	69.4	nucl: 13, cysk: 1
BcARR11-b	1449	483	5.38	54.1	nucl: 9, chlo: 2, extr: 1, E.R.: 1, cysk: 1
BcAPRR4-a	1323	441	5.93	50.6	nucl: 8, chlo: 2, cyto: 2, pero: 1, cysk: 1
BcAPRR4-b	1527	509	5.35	58.9	nucl: 11, cyto: 2, chlo: 1
BcARR12	1392	464	5.97	51.1	nucl: 13, cysk: 1
BcARR21-a	2685	895	5.57	98.0	nucl: 12, pero: 1, cysk: 1
BcARR21-d	1803	601	5.23	67.8	nucl: 9, chlo: 2, cyto: 1, plas: 1, cysk: 1
BcARR21-b	1785	595	5.80	67.3	nucl: 11, chlo: 2, plas: 1
BcARR21-c	1383	461	9.50	51.9	nucl: 12, pero: 1, cysk: 1
